# The association between Lymphogranuloma venereum and HIV among men who have sex with men: systematic review and meta-analysis

**DOI:** 10.1186/1471-2334-11-70

**Published:** 2011-03-18

**Authors:** Minttu M Rönn, Helen Ward

**Affiliations:** 1 Department of Infectious Disease Epidemiology, Imperial College London, Old Medical School, St. Mary's Campus, Norfolk Place, Paddington, W2 1PG London, UK

## Abstract

**Background:**

Lymphogranuloma venereum (LGV) is an important re-emerging sexually transmitted infection which is reported to affect particularly HIV-positive men who have sex with men (MSM). The aim of this study is to quantify the association between LGV and HIV in the context of the current emergence of LGV.

**Methods:**

A systematic review was performed on the emergence of LGV among MSM since 2000. We report the prevalence of HIV infection from descriptive studies of MSM with LGV, and conduct a meta-analysis to produce a summary estimate of the association between LGV and HIV from case-control studies where cases were MSM with LGV and controls were MSM with rectal chlamydia caused by non-LGV serovars.

**Results:**

The prevalence of HIV among LGV cases ranges from 67% to 100% in 13 descriptive studies. There is a significant association between HIV and LGV (odds ratio 8.19, 95% CI 4.68-14.33).

**Conclusions:**

HIV-positive MSM are disproportionately affected by LGV highlighting the importance of prevention efforts to be targeted to this group. Further research is needed to determine whether the association is due to biological or behavioural factors.

## Background

Lymphogranuloma venereum (LGV) is a sexually transmitted infection (STI) which largely disappeared from the Western world after the introduction of antibiotics. LGV is caused by the species *Chlamydia trachomatis *(CT) which produces infection in humans and includes three biovars [1]; serovars A-C cause trachoma, an eye infection that mainly spreads via child-to-child transmission and is the leading cause of preventable blindness, while the second biovar (serovars D-K, and occasionally also B [2]) is transmitted sexually [3]. Serovars A-K have a limited host-cell range, they infect mainly squamocolumnar epithelial cells and are incapable of infecting deeper tissues. The third biovar, LGV, is formed by serovars L1, L2 and L3 and it causes a more invasive disease due to its ability to infect macrophages and consequently it can spread to lymphatic tissue at the site of infection leading to a systemic disease [4]. LGV is classified as a genital ulcer disease (GUD) without clear tissue tropism for mucosa contrary to non-LGV chlamydia, and it has been considered endemic in the tropics although LGV epidemiology in these areas has not been systematically described. In 2003 a cluster of LGV cases was reported in Rotterdam, the Netherlands [5] and, following an international alert, case reports were published of similar outbreaks in large European cities [6-10]. Reports of LGV from the United States [11], Canada [12] and Australia [13] followed.

The clinical features of LGV have historically been divided into three stages: the primary stage involves the site of inoculation where a small papule, ulcer, herpetiform lesion or nonspecific urethritis appears after incubation period of 3-30 days [4]. During the secondary stage, which emerges after 10-30 days or even months later, local lymph nodes draining the site of primary infection enlarge and necrotic areas develop following an inflammatory process which results in chronic oedema and formation of ulcers and sclerosing fibrosis. Without adequate treatment a tertiary phase with chronic inflammatory response will follow. This is characterized by genital ulcers, fistulas, rectal strictures and genital elephantiasis. The scarring and formation of fibrotic tissue often requires surgical repair [4]. The classical manifestation in men is characterised by inguinal lymphadenopathy. During the recent emergence of LGV, the clinical manifestation has been different. Acute ulcerative proctitis or proctocolitis is often seen as the primary manifestation [4,14] (approximately 96% of the cases in the United Kingdom [15]) while inguinal lymphadenopathy alone has been observed in only a few cases [16]. Rectal discharge, pain, bleeding [14] and systemic symptoms such as malaise [17] and high white blood cell count (>10/high power field) in gram stained rectal smears [18] have been reported as indicators of LGV. It is not clear why inguinal symptoms, indicating urethral acquisition of LGV, have been so rare and it has been suggested that different modes of transmission, such as sexual practices of fisting and use of sex toys and other fomites, are contributing to the spread of LGV. Traumatic practices to the mucosa have been previously associated with the acquisition of other rare STIs such as sexually acquired hepatitis C virus [19].

In response to the outbreak surveillance systems were established in several affected countries. These have produced a consistent picture of cases among men who have sex with men (MSM), many of whom are co-infected with HIV (for example 74% co-infection rates in the UK [20] and over 70% reported in other European countries [21]). On average, affected patients have also been older (mean age above 35 [21]) than MSM presenting to STI clinics for other reasons, and with a high rate of concurrent STIs and hepatitis C (14% of cases in the UK [20]). In 2009 a case-finding study in genitourinary medicine (GUM) clinics in London found a prevalence estimate of LGV among MSM to be 1.2% (36 out of 3076 men) [22] while another case-finding exercise conducted in four GUM clinics in London and Brighton found LGV positivity among rectal samples from MSM to be 0.9% (61 of 6778 rectal samples tested for LGV) [23].

As LGV seems to be circulating mainly among HIV-positive MSM it is important to consider the effect these STIs have on each other [24]. STIs may contribute to transmission of HIV and concurrent HIV infection may alter the epidemiology of an STI: both STI and HIV can become more infectious in a co-infected individual. HIV may also increase the susceptibility of an HIV-positive individual to other STIs and HIV may be transmitted more easily in discordant couple if the HIV-negative partner has a concurrent STI [25]. In meta-analysis by Rottingen *et al. *(2001) [25] both GUD and CT were found to increase susceptibility to HIV-infection in heterosexual transmission and it would seem plausible that LGV may increase HIV transmission also in homosexual partnerships. The aim of this study is to quantify the association between HIV infection and LGV among MSM in the context of the current emergence of LGV.

## Methods

### Search strategy

A systematic review was conducted using PubMed of the National Center for Biotechnology Information (NCBI), MEDLINE of the National Library of Medicine (NLM) and Web of Science (the latter two via ISI Web of Knowledge platform). The search was restricted for the years 2000-2009. The searches were performed on 17 June 2009 using the search words "Lymphogranuloma venereum" (MeSH, and topic search) and the acronym LGV with Boolean search looking for any of the words. No restrictions were selected on the type of studies, or publication language. An additional search was done in Eurosurveillance [26] on 18 June 2009 using the advanced search and selecting "Lymphogranuloma venereum - LGV" from the subject list. The article search focused on covering all scientific publications of LGV after 2000, as case-finding and reporting of the emergence of LGV has started after 2003. Novel diagnostic methods have been developed recently [27] and this has enhanced the reliability of LGV diagnosis.

The grey literature was searched online using the websites of national public health agencies and professional organisations including the Health Protection Agency of England and Wales, the British Association for Sexual Health and HIV, the Public Health Agency of Canada and the Centres for Disease Prevention and Control of the United States. Reference lists of LGV publications were utilised to identify relevant literature and they were also reviewed for completeness of already found publications. No attempt was made to identify unpublished studies. Ethical approval was not sought, since the study relied on published data only.

### Study selection

The study selection was done in two stages: during the first phase all publications involving a component of LGV epidemiology or management of LGV were included apart from publications focusing on the molecular biology of LGV or CT which were excluded. The study selection at this point was done based on abstract or the full publication if abstract did not give sufficient information. At the second phase complete publications were reviewed and their suitability in respect to the research objective was assessed. Studies were included if they met the following criteria: the main outcome was LGV and the study population was composed of MSM with data available for the level of HIV co-infection among study subjects. The selection of papers was restricted to MSM as they have been most affected by LGV in developed countries, and because the clinical manifestation of LGV is different among homosexuals than heterosexuals. Case-series and conference abstracts were excluded at the second stage of the review.

### Data extraction

From the descriptive studies that met all the inclusion criteria, the following variables were obtained: first author, year of publication, purpose and type of the study, study period, sample size, number of confirmed LGV cases (confirmation of LGV status requires detection of L1-L3 serovars from a chlamydia positive sample), age as reported in the paper, number of HIV positive LGV cases as well as number of cases with negative or unknown HIV status. Additionally from case-control studies information on the effect sizes and their confidence intervals and two by two table of the distribution of HIV serostatus was retrieved. When several publications reported data from the same setting, the latest or most complete report was used. The eligible publications were in English and Swedish as the first author is fluent in both languages.

### Analytic methods

To estimate the prevalence of HIV among LGV cases, raw pooled prevalence estimates were calculated based on the numbers of HIV-positive and negative individuals given in the publications. A summary estimate was constructed and heterogeneity of the estimates explored using meta-analysis (using inverse-variance and derSimonian and Laird method [28]). To calculate variance estimates, studies were excluded if the number of expected cases was less than 5 in order to avoid skewed summary estimates. In order to make the distribution nearer to normal meta-analysis was run using logits (log of the odds) of the prevalence estimates and final results transformed back to prevalence [29].

To analyse LGV-HIV association in case-control studies, a meta-analysis was performed using Mantel-Haenszel fixed-effect method as presented by Petitti (2000) [30]. For tests of heterogeneity chi-square test was used and also random-effect method (derSimonian-Laird method) which yields close to identical results to fixed-effect method in the absence of heterogeneity. Statistical analyses were done using STATA10. Reporting followed the guidance of PRISMA statement (preferred reporting items for systematic reviews and meta-analyses) [31].

## Results

### Details of included and excluded articles

The search produced the following results: PubMed 270, Medline 273 and Web of Science 264. Many citations were common to all three search engines and after removal of duplicates there was a total of 368 original publications for the years 2000 to 2009. Eurosurveillance had 21 publications under the subject heading Lymphogranuloma venereum out of which 3 were identified through Eurosurveillance search only[5,8,10]. Searching the grey literature gave 4 additional LGV-related results [32-35] and a search of the reference lists gave six more publications [36-41] that fulfilled the primary inclusion criteria.

**Figure 1:**
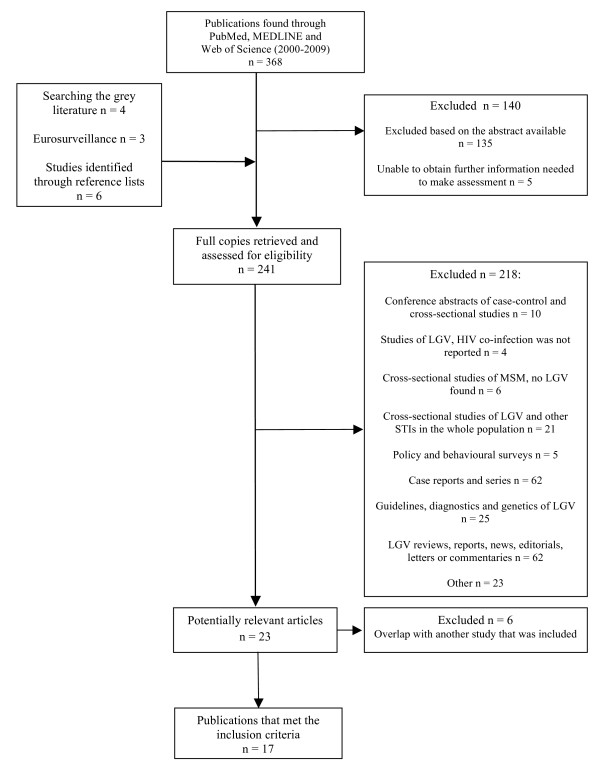
**Flow chart for study selection and process**.

During the first stage of the review 140 publications were excluded (Figure 1). Full copies of 241 articles were reviewed in the second stage of the review. The systematic review identified 23 potentially relevant articles from which data extraction was performed and after excluding 6 publications with overlapping study period [15,17,42-45] 17 publications remained that fulfilled the inclusion criteria

### Estimating level of HIV co-infection among LGV-cases

The review identified 13 cross-sectional or descriptive studies that provided information on the prevalence of HIV among LGV cases. The majority were surveillance reports of LGV cases, and characteristics are presented in Additional file 1, Table S1. A total of 1145 confirmed LGV cases were reported by the studies, of which 985 had epidemiological data (86.3% of cases). Among the confirmed cases there was a high level of HIV co-infection, ranging from 67% to 100% across the reports. The raw pooled prevalence estimate was 77.9% (95% CI 75.0-80.8%) among cases with known HIV status. However, there was a significant amount of missing data on HIV status, with 19.8% of cases having unknown status. To statistically explore heterogeneity, a meta-analysis for the prevalence estimates was performed using the known HIV statuses from the publications. This was done for five studies with expected number of cases of five or more [20,46-49] (the eligible publications are highlighted in table S1). A fixed-effect model gave a summary estimate of 74.5% and random-effect model 78.2% (raw pooled estimate of the included five studies 76.3%). The test for heterogeneity among these studies was statistically significant (p-value < 0.001) and thus a summary estimate was deemed an inappropriate approach and should be used for illustrative purposes only due to the underlying differences between studies.

### Estimating the association between LGV and HIV

**Figure 2:**
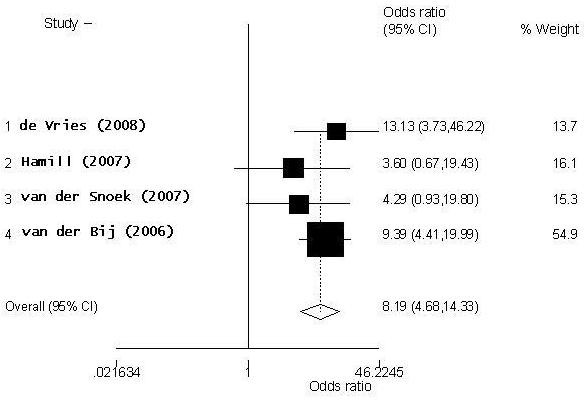
**Forest plot of HIV-LGV association using fixed-effect model**.

Four case-control studies were indentified that estimated the association between LGV and HIV (Additional file 1, Table S2). In these studies all the cases have LGV proctitis and included a control group with non-LGV CT (some studies had more than one control group). A summary OR for the HIV-positives was calculated using HIV-seronegative patients as a reference group, and the resulting forest plot is presented in Figure 2. The summary estimate demonstrates that MSM with LGV are eight times more likely to have HIV (OR 8.19, 95% CI 4.68-14.33, p-value < 0.001) compared to MSM with non-LGV CT. The statistical test for heterogeneity did not show a significant difference between the study groups (p-value 0.519). Study by van der Bij has the largest weight as it has over twice as many cases and controls compared to other studies. Due to the small number of people with an unknown HIV-serostatus (only van der Bij had a large number of participants with unknown status), the association between that and LGV was not explored.

As a random-effect model produced very similar results to a fixed-effect model, further statistical tests for heterogeneity were not conducted. Sources of heterogeneity were explored by looking into potential differences in the study design. There seems to have been few methodological differences between the studies: all the study participants were recruited on STI clinics based on their sexual risk-behaviour or symptoms reported. All the LGV cases were confirmed using either real-time PCR of RFLP-PCR as were non-LGV CT controls. It is unlikely that there are significant differences between the study populations as three of the studies were conducted in the Netherlands (two in Amsterdam and one in Rotterdam) and one study in London, UK. Therefore it seems that no large methodological, clinical or geographic sources of heterogeneity can be detected between the studies.

## Discussion

We found consistent evidence of a strong association between HIV and LGV across the literature. In thirteen descriptive studies at least two-thirds of MSM with LGV were co-infected with HIV. In a meta-analysis, MSM with LGV were over eight times more likely to have HIV (OR 8.19, 95% CI 4.68-14.33) than those who had non-LGV chlamydia infection. This association is stronger than for other sexually transmitted infections which have also re-emerged in MSM over the past decade [50]. As the descriptive studies presented results mainly in aggregate form, it was not possible to further investigate the effect of LGV genotype or rectal and non-rectal infection on the association between HIV and LGV. However, in the majority of studies rectal symptoms and proctitis were the dominant manifestation of LGV, as shown by results from the three largest studies: in the UK 90% of confirmed cases had proctitis [20], in France only rectal swabs were included in the surveillance system [47], and 91% of cases in Netherlands had proctitis [49]. Similarly only aggregate information was available for LGV genotype, but Canada [12], France [47] and the Netherlands [46] found only L2b genotype in the samples that were sequenced. Other descriptive studies selected for this study found mainly L2 serotype with predominantly L2b variant in the sequenced samples (such as 426/470 of samples in UK surveillance that were confirmed L2b [20]). Primary data sources would be needed to analyse differences between HIV positive and HIV negative LGV cases.

A number of detailed reviews have been published on the emergence of LGV [14,51]. However to our knowledge this review is the first to systematically identify and quantitatively analyse the association between HIV and LGV in the re-emergence of LGV, and includes data from 17 reports. However, it is limited by the inclusion of a number of surveillance reports. Surveillance of LGV is said to suffer from the lack of consistency between countries giving rise to different case-definitions and surveillance methods, some of which are based on voluntary and potentially partial reporting [52]. Surveillance reports formed a large proportion of publications used for the HIV-prevalence estimates, but the studies in this review all defined detection of LGV serovars as a requirement for confirmed cases. Nevertheless it is possible that cases have been identified with different accuracy between countries and the international data may not be comparable. Previous literature has noted that the variation in the structure of surveillance systems limits cross-country comparison [53]. The geographical differences may be a result of variation in sexual partnerships, differential sexual behaviours (such as seroadaptive behaviour), testing and case finding policies, time of introduction of LGV into the network and the impact of local public health interventions [50].

In this review we did not apply strict quality criteria. This is due to the relatively small number of published studies and our desire to be inclusive in order to increase sample size. This means that we cannot exclude the possibility that sampling and measurement differences may account for some of the heterogeneity. Outcome-level assessment for LGV was performed by including only confirmed cases (identification of LGV serovars required for a confirmed case) which assures the validity of the data for the main outcome. Due to the small number of studies conducted to date and the recent emergence of LGV, publication bias was not thought to be relevant in this analysis. The search and article retrieval was conducted by the first author alone, but the analysis and interpretation was done by both authors.

The strong association between LGV and HIV in an unadjusted model is interesting, and some association would be expected to remain after adjusting for confounders, especially since the association is strong even though the control group represents another high risk group of MSM with a non-LGV CT infection. It is possible that there may be a biological interaction between HIV-infection and LGV. HIV-infection has been reported to cause abnormalities in the structure and function of the gastrointestinal tract as well as abnormal lymphocyte trafficking [54] that may facilitate LGV acquisition. In a South African cohort of patients with GUD, LGV infection was diagnosed more often among the HIV-infected participants compared to HIV-negative participants with relative risk 1.3 (95% CI 1.2-1.4) [55]. Also in the Bahamas, an LGV epidemic was associated with crack cocaine use and HIV infection [56]. Two of the case-control studies included in this review compared median CD4 count between cases and controls, but neither found a significant difference [57,58]. van der Bij *et al. *[18] also hypothesised that immunorestoration inflammatory syndrome could explain the emergence of this highly symptomatic LGV. They analysed the association between having symptomatic LGV and start of highly active antiretroviral therapy (HAART) but no association was found. Time-series analysis may be a more effective way to measure the potential interaction between HIV and LGV.

The strongest confounder for this hypothesis is high-risk sexual behaviour. There have been behavioural changes in MSM communities as a response to HIV epidemic, and serosorting may make HIV-positivity an intermediating factor: individuals participate in certain type of practices partly because of their HIV-infection [59]. Seroadaptive behaviour can reduce the number of new HIV infections but it also facilitates the spread of other STIs and can create dense sexual networks where disease propagation is fast [53]. Exploring this further was not possible in this review because of the lack of comparable data on sexual behaviour.

## Conclusions

The findings from HIV prevalence estimates and case-control studies reveal that HIV-infected MSM are disproportionately affected by the emergence of LGV. LGV prevention efforts should be targeted towards HIV-infected sexually active MSM. Although the majority of LGV infected individuals are HIV-positive, a proportion of diagnosed LGV cases are HIV-negative and this group would benefit from primary HIV prevention as they belong to sexual networks where HIV-seropositivity is common and subsequently they are at high risk of HIV-infection.

The strong association between LGV and HIV co-infection identified from case control studies may indicate a biological synergy between LGV and HIV, but further research is required to explore the relationship between HIV-infection, behaviour and acquisition of LGV.

## Competing interests

The authors declare that they have no competing interests.

## Authors' contributions

HW and MR defined the research questions. MR performed the review and conducted the analysis and HW reviewed the results. Both authors wrote or reviewed the article. Both authors read and approved the final manuscript.

## Pre-publication history

The pre-publication history for this paper can be accessed here:

http://www.biomedcentral.com/1471-2334/11/70/prepub

## Supplementary Material

Additional file 1 **Table S1 and Table S2**. Table S1 presents the descriptive studies selected for the analysis of prevalence of HIV co-infection in LGV cases, and Table S2 presents the case-control studies with estimates of association between HIV-positivity and LGV [60-67]. Click here for file
