# Supplementary material for: The association between Lymphogranuloma venereum and HIV among men who have sex with men: systematic review and meta-analysis
Source: BMC Infect Dis. 2011 Mar 18;11:70. doi: 10.1186/1471-2334-11-70 (PMC3070636; doi:10.1186/1471-2334-11-70)
Supplement: Additional file 1 — Table S1 and Table S2. Table S1 presents the descriptive studies selected for the analysis of prevalence of HIV co-infection in LGV cases, and Table S2 presents the case-control studies with estimates of association between HIV-positivity and LGV [60-67]. [file 1471-2334-11-70-S1.DOCX]

| **Table 1. Descriptive studies selected for analysis of the prevalence of HIV co-infection in LGV cases** | | | | | | | | | | |
| --- | --- | --- | --- | --- | --- | --- | --- | --- | --- | --- |
| **First author**  **[Reference]** | **Location** | **Aim of the study** | **Study design** | **Study period** | **Population** | **Sample size** | **Confirmed LGV** | **Age:**  **[mean]/ median** **/(range)** | **HIV+ (% of those with known HIV status)** |  |
| **Velicko, I. 2009 [**[**60**](#_ENREF_60)**]** | Sweden | Describe the Swedish LGV situation | Descriptive, surveillance | 2007 | Reported LGV, MSM | 15 LGV cases | 15 | 38 (23–58) | 11/13  (84.6 %) |  |
| **Stary, G. 2008 [**[**61**](#_ENREF_61)**]** | Vienna,  Austria | Occurrence of LGV in Vienna | Cross-sectional | 11.2005 – 11.2006 | Symptomatic MSM | 24 men with symptoms indicative of LGV | 15 | [41,3] | 11/13  (84.6 %) |  |
| **Cusini, M. 2008 [**[**62**](#_ENREF_62)**]** | Italy | Describe the Italian LGV situation | Descriptive | 3.2006 – 12.2007 | Identified LGV cases, MSM | 13 LGV cases | 12 | n/a | 8/12  (66.7 %) |  |
| **Vall Mayans, M. 2008 [**[**63**](#_ENREF_63)**]** | Barcelona, Spain | Describe the LGV situation in Barcelona | Descriptive, surveillance | 9.2007 –  4.2008 | Reported LGV, MSM | 21 LGV cases | 21 | n/a | 19/21  (90.5 %) |  |
| **Jebbari, H. 2007 [**[**20**](#_ENREF_20)**]** | London, UK | Describe the British LGV situation | Descriptive, surveillance | 10.2004 – 4.2007 | LGV cases, mostly MSM | 3094 specimens processed, 492 with LGV; 423 with enhanced surveillance data | 492 | 40 (24–69) | 312/423  (73.8 %) |  |
| **Stark, D.**  **2007 [**[**64**](#_ENREF_64)**]** | Australia | Occurrence of LGV in high risk population | Cross-sectional, surveillance | 10.2005 –  7.2006 | Rectal swabs from MSM submitted for CT testing in a hospital | CT positive samples (n = 29) tested for LGV | 4 | [49.5] (43–55) | 4/4  (100 %) |  |
| **Pathela, P. 2007 [**[**48**](#_ENREF_48)**]** | New York State, USA | Describe the LGV situation in the State of New York | Descriptive, surveillance | 5.2005 –  4.2006 | Rectal swabs sent for LGV testing | 168 rectal swabs from men | 31 | 33 | 26/31  ( 83.9%) |  |
| **Bremer, V. 2006 [**[**65**](#_ENREF_65)**]** | Germany | Describe the German LGV situation | Descriptive, surveillance | 1.2003 – 11.2005 | LGV cases | 61 confirmed cases, also 17 probable and possible | 61 | [39] | 42/44  (95.5 %) |  |
| **van de Laar, M. 2006 [**[**49**](#_ENREF_49)**]** | Netherlands | Describe the Dutch LGV situation | Descriptive, surveillance | 1.2004 – 1.2006 | LGV cases | 179 confirmed cases, 104 with enhanced surveillance data | 179 | [40] (26–58) | 70/88  (79.5 %) |  |
| **Spaargaren, J. 2005 [**[**46**](#_ENREF_46)**]** | Amsterdam, NL | Analyse symptoms of LGV and non-LGV patients | Retrospective cross-sectional | 2002-2003 | MSM treated at an STD clinic | 74 rectal CT samples, 45 with L2 | 45 | 45 (25.9-47.6) | 31/45  (68.9 %) |  |
| **Herida, M. 2006 [**[**47**](#_ENREF_47)**]** | France | Describe the French LGV situation | Descriptive, surveillance | 3.2004 – 12.2005 | MSM with rectal CT | 328 CT positive rectal samples | 244 | 39 (34–44) | 82/96  (85.4 %) |  |
| **Kropp, R. 2005 [**[**12**](#_ENREF_12)**]** | Canada | Describe the Canadian LGV situation | Descriptive, surveillance | By 5.2005 | Reported LGV cases | 22 reported, 19 with surveillance data | 16 | (29–47) | 8 (n/a) |  |
| **Gebhardt, M.2005 [**[**66**](#_ENREF_66)**]** | Switzerland | Retrospective analysis of LGV in Switzerland | Cross-sectional | 1.1999 –  5.2005 | MSM testing for rectal chlamydia | 35 rectal samples | 10 | (25–56) | 7/10  (70%) |  |

| **Table 2. Case-control studies with estimates of the association between HIV and LGV** | | | | | | | | | | |  |
| --- | --- | --- | --- | --- | --- | --- | --- | --- | --- | --- | --- |
| **First author [reference]** | **Location** | **Aim of the study** | **Study design** | **Study period** | **Population** | **Cases** | **Controls** |  | **Unadjusted effect estimate** | **Adjusted effect estimate ^(*^** | |
| **de Vries, H.J.C*.***  ***et al.***  **(2008)**  **[**[**67**](#_ENREF_67)**]** | Amsterdam | Estimate risk  factors  associated  with LGV  proctitis | Case-control | 8.2004 – 8.2005  (LGV cases until 4.2006) | MSM  presenting  at STI  clinic  with  proctitis | 32 LGV proctitis | **a)** 30 non-LGV CT proctitis  **b)** 22 gonorrhoea proctitis  **c)** 41 proctitis of unknown aetiology | HIV+ | **(a)**  13.1 (3.7-46.2) |  | |
| **Hamill, M.**  ***et al.***  **(2007)**  **[**[**57**](#_ENREF_57)**]** | London | To assess  behavioural and  clinical  features  of LGV and  non-LGV CT  proctitis | Case-control | 4.2004 – 6.2005 | MSM with symptomatic CT proctitis presenting to a GUM clinic | 32 LGV with rectal symptoms | 31  non-LGV CT with rectal symptoms | HIV+ | 3.6 (0.67-19.4) |  | |
| **Van der Snoek, E.M.**  ***et al.***  **(2007)**  **[**[**58**](#_ENREF_58)**]** | Rotterdam, | To assess the usefulness  of serological titres in diagnostics  of LGV | Case-control | 5.2003 – 11.2005 | MSM presenting at STI clinic with proctitis | 24 LGV proctitis | 15  non-LGV CT proctitis | HIV+ | 4.3 (0.9-19.8) |  | |
| **Van der Bij, A.K.  *et al.***  **(2006)**  **[**[**18**](#_ENREF_18)**]** | Amsterdam | To assess risk factors and clinical predictors  of LGV | Retro-spective  case-control | 2002-2003 | All CT positive rectal samples from MSM | 87 LGV proctitis | **a)** 377 non-LGV CT proctitis  **b)** 2677 MSM reporting unprotected anal intercourse without CT | HIV –HIV+  Unknown | **(a)**  1   - 1. (4.4-20.0)   2.8 (1.3-6.2) | **(a)**  1  5.7 (2.6 - 12.8)  2.7 (1.2-6.2) | |
| *) Adjusted effect estimates:  Van der Bij *et al* (2006) adjusted for risk factors that were chosen by backward selection (variables adjusted for were not reported in the paper).  In the paper by de Vries *et al.* (2008) HIV was no longer statistically significant in multivariate analysis (only significant results presented in the paper).  Hamill *et al.* (2007) and van der Snoek *et al.* (2007) did not include multivariate analysis in their methods. | | | | | | | | | | |  |
